# Serum immune mediators as novel predictors of response to anti-PD-1/PD-L1 therapy in non-small cell lung cancer patients with high tissue-PD-L1 expression

**DOI:** 10.3389/fimmu.2023.1157100

**Published:** 2023-05-15

**Authors:** Afsheen Raza, Reyad Mohsen, Aladdin Kanbour, Abdul Rehman Zar Gul, Anite Philip, Suma Vijayakumar, Shereena Hydrose, Kirti S. Prabhu, Aisha Khamis Al-Suwaidi, Varghese Philipose Inchakalody, Maysaloun Merhi, Dina M. Abo El-Ella, Melissa Annrose Tauro, Shayista Akbar, Issam Al-Bozom, Wafa Abualainin, Rajaa Al-Abdulla, Shaza Abu Sirriya, Suparna Hassnad, Shahab Uddin, Mohamed Izham Mohamed Ibrahim, Ussama Al Homsi, Said Demime

**Affiliations:** ^1^ Department of Medical Oncology, National Center for Cancer Care and Research, Hamad Medical Corporation, Doha, Qatar; ^2^ Translational Cancer Research Facility, Translational Research Institute, Academic Health System, Hamad Medical Corporation, Doha, Qatar; ^3^ Translational Research Institute (TRI), Academic Health System, Hamad Medical Corporation, Doha, Qatar; ^4^ Department of Human Genetics, Sidra Medical and Research Center, Doha, Qatar; ^5^ College of Health and Life Sciences, Hamad Bin Khalifa University, Doha, Qatar; ^6^ Department of Laboratory Medicine and Pathology, Hamad Medical Corporation, Doha, Qatar; ^7^ Diagnostic Genomic Division , Department of Laboratory Medicine and Pathology, Hamad Medical Corporation, Doha, Qatar; ^8^ Department of Radiation Oncology, National Center for Cancer Care and Research, Hamad Medical Corporation, Doha, Qatar; ^9^ Translational Research Institute and Dermatology Institute, Academic Health System, Hamad, Medical Corporation, Doha, Qatar; ^10^ Laboratory Animal Research Center, Qatar University, Doha, Qatar; ^11^ Clinical Pharmacy and Practice Department, College of Pharmacy, Qatar University (QU) Health, Qatar University, Doha, Qatar

**Keywords:** non-small cell lung cancer, anti-PD-1, anti-PD-L1, tissue PD-L1, predictive soluble biomarkers, CEA

## Abstract

Non-small cell lung cancer (NSCLC) is the leading cause of cancer-related morbidity and mortality worldwide. Immune checkpoint inhibitors (ICIs) including anti-PD-1 and anti-PD-L1 antibodies, have significantly changed the treatment outcomes with better overall survival, but only 15-40% of the patients respond to ICIs therapy. The search for predictive biomarkers of responses is warranted for better clinical outcomes. We aim here to identify pre-treatment soluble immune molecules as surrogate biomarkers for tissue PD-L1 (TPD-L1) status and as predictors of response to anti-PD-1/PD-L1 therapy in NSCLC patients. Sera from 31 metastatic NSCLC patients, eligible for anti-PD-1/PD-L1 or combined chemoimmunotherapy, were collected prior to treatment. Analysis of soluble biomarkers with TPD-L1 status showed significant up/down regulation of the immune inhibitory checkpoint markers (sSiglec7, sSiglec9, sULBP4 and sPD-L2) in patients with higher TPD-L1 (TPD-L1 >50%) expression. Moreover, correlation analysis showed significant positive linear correlation of soluble PD-L1 (sPD-L1) with higher TPD-L1 expression. Interestingly, only responders in the TPD-L1 >50% group showed significant down regulation of the immune inhibitory markers (sPD-L2, sTIMD4, sNectin2 and CEA). When responders vs. non-responders were compared, significant down regulation of other immune inhibitory biomarkers (sCD80, sTIMD4 and CEA) was recorded only in responding patients. In this, the optimal cut-off values of CD80 <91.7 pg/ml and CEA <1614 pg/ml were found to be significantly associated with better progression free survival (PFS). Indeed, multivariate analysis identified the cutoff-value of CEA <1614 pg/ml as an independent predictor of response in our patients. We identified here novel immune inhibitory/stimulatory soluble mediators as potential surrogate/predictive biomarkers for TPD-L1 status, treatment response and PFS in NSCLC patients treated with anti-PD-1/PD-L1 therapy.

## Introduction

Lung Cancer is the second most common cancer and a leading cause of cancer-related deaths (a total of 18% of cancer deaths) worldwide. In 2020, 2.2 million new cancer cases and 1.8 million deaths were reported for lung cancer. The 5-year survival rate is poor, ranging between 10-20% in developed countries (1).

Non-small cell lung cancer (NSCLC) is the most common cancer type, accounting for approximately 85% of lung cancer cases (2). Treatment management includes surgical removal, adjuvant chemotherapy, radiotherapy, and molecular-targeted therapies for patients with driver mutations (3). However, in a cohort of metastatic NSCLC patients with wild type epidermal growth factor receptor (EGFR), anaplastic lymphoma kinase (ALK) gene and tumor tissue expressing programmed death ligand-1(TPD-L1), treatment mainly comprises of FDA approved immune checkpoint inhibitors (ICIs), anti-programmed death protein 1/programmed death ligand 1 (PD-1/PD-L1) (4). ICIs are mainly monoclonal antibodies that target immune checkpoints, PD-1, and PD-L1 and block their pathways to help unleash a robust anti-tumor response [5]. Although ICIs have been shown to improve the overall survival in NSCLC patients, limited response rates, ranging between 15-40%, have been documented (5). Several intrinsic and extrinsic factors circulating within the host tumor microenvironment such as regulatory T cells (T regs), myeloid derived suppressor cells (MDSCs), M2 macrophages, immune checkpoints, cytokines and chemokines, have been associated with manipulation of immune response to facilitate tumor progression (6). On the other hand, it is postulated that soluble forms of immune checkpoint T and Natural killer (NK) cell receptors/ligands such as soluble programmed death protein 1 (sPD-1), soluble programmed death ligand 1 (sPD-L1), soluble programmed death ligand 2 (sPD-L2), soluble T cell immunoglobulin domain and mucin domain 3 (sTIM3), soluble UL16 binding protein 1/4 (sULBP-1/4), soluble Natural killer group 2D receptor and ligands sNKG2DL may affect treatment dynamics, either in an immune inhibitory or immune stimulatory manner (7–9). Some of the immune modulatory mechanisms associated with soluble forms include their binding to the treatment active site to hinder treatment efficacy, activation of immune suppressive molecules, inhibition of Interleukin-2 (IL-2) production/T cell activation, T cell apoptosis, upregulation of Tumor necrosis factor-α (TNF-α)/Interferon-gamma (IFN-γ) and early activation of CD8^+^ T cells leading either to tumor immune escape or control (10–13). In anti-PD-1/PD-L1 treated NSCLC patients, a limited number of studies have associated soluble immune checkpoint markers with prognosis, response to treatment, and overall survival (14–19). The results from these studies indicate a potential role of soluble immune checkpoint mediators as biomarkers for patient stratification (responding vs. non-responding patients) and treatment dynamics. However, most studies have focused mainly on sPD-1 and sPD-L1, indicating a lack of data on other soluble T and NK immune checkpoint markers and their role in prognosis or prediction of response.

In addition to soluble T and NK markers, several studies have also reported on the role of tumor secreted antigens, such as Carcinoembryonic Antigen (CEA), Cytokeratin Fragment 19 (CYFRA21-1), and Carbohydrate Antigen 125 (CA-125), as biomarkers in some tumor types (20–22). These soluble antigens are expressed in various cancers, and some of them are widely used for clinical assessment and treatment monitoring in chemotherapyIn ICI-treated patients, limited number of studies have documented the role of circulating tumor antigens as dynamic biomarkers (23–27). However, the utility of these biomarkers in assessing immunotherapy efficacy in NSCLC patients is still poorly explored, indicating a significant knowledge gap on their role as potential predictive/prognostic biomarkers.

In addition to soluble biomarkers, tissue markers have also been reported as predictors of response. To date, TPD-L1, measured by the immunohistochemistry (IHC) technique, is the only predictive marker approved by FDA as a companion diagnostic for anti-PD-1 antibody treatment in advanced NSCLC. To date, several randomized controlled trials have associated various TPD-L1 tumor proportional scores (TPS) such as ≥1%, ≥5%, ≥10%, and ≥50% with clinical efficacy endpoints such as overall survival (OS), progression-free survival (PFS) and objective response rate (ORR) (28, 29). However, conflicting data regarding the utility of TPD-L1 TPS has been reported, with some trials reporting it as a powerful predictive marker for OS while others indicate limited value of this marker (30–33). In lieu of this, limited studies have investigated the linear relationship of TPD-L1 expression with soluble biomarkers and clinical response in ICI-treated NSCLC patients to understand the role of soluble mediators as surrogate markers for TPD-L1 (34–36). This is an essential area of research since finding non-invasive surrogate markers for tissue can have various advantages, such as ease of sampling, longitudinal monitoring, and limited heterogeneity.

Pre-treatment assessment of dynamic biomarkers is an essential timeline as it helps understand the correlation of baseline biomarkers with disease/treatment dynamics (37, 38). It is well documented that early markers of response can serve as powerful tools for patient stratification and prediction of response (39–41). For ICI-treatment in NSCLC patients, the significance of pre-treatment biomarkers is of utmost importance as this cohort of patients has limited treatment options, and early response prediction can facilitate better patient management.

We aimed here to identify pre-treatment soluble immune checkpoint and circulating tumor antigens as surrogate/predictive markers in TPD-L1 expressing patients and to determine the role of soluble markers as predictors of response in anti-PD-1/PD-L1 treated NSCLC patients.

## Methods

### Study population and data collection

This prospective study was conducted at the National Center for Cancer Care and Research (NCCCR), Hamad Medical Corporation (HMC), Doha, Qatar, from September 2020 to July 2022. A total of 31 metastatic advanced-stage NSCLC patients eligible for treatment with anti-PD-1 (Nivolumab, Pembrolizumab), anti-PD-L1 (Durvalumab) monotherapy or combined chemoimmunotherapy (Carboplatin + Pemetrexed + Pembrolizumab) were enrolled in the study. Demographics and clinical characteristics of all patients, including age, gender, ethnicity, smoking history, histology, stage, differentiation status, Eastern Cooperative Oncology Group performance status (ECOG PS), genetic aberrations, Tissue PD-L1 expression, metastasis sites, previous lines of radiotherapy/chemotherapy, imaging and clinical response were extracted from electronic health record system of HMC (CERNER^®^).

Written informed consent was obtained from all eligible participants per Declaration of Helsinki and good clinical practice guidelines. The study was approved by the Institutional Review Board of HMC (MOPH-HMC-020).

### Sample collection

Blood sample (10 ml) was collected from eligible patients before anti-PD-1/anti-PD-L1 monotherapy or combined chemoimmunotherapy treatment in BD Vacutainer SST II Advance Serum tubes (Becton Dickenson, USA). The tubes were centrifuged at 1300 g for 10 minutes and the extracted serum was cryopreserved at -80°C until further analysis.

### Measurement of soluble immune checkpoint mediators and circulating tumor biomarkers

According to manufacturers’ instruction, the level of soluble immune checkpoint T and NK cell mediators was detected using the Immuno-Oncology Checkpoint 14-Plex Human ProcartaPlex Panel 1, Panel 2, and Immuno-Oncology Checkpoint 9-Plex Human ProcartaPlex Panel 3 (ThermoFisher Scientific, USA). The 37 analytes tested included CD27, CD28, 4-1BB, GITR, HVEM, BTLA, CD80, CTLA-4, IDO, LAG-3, PD-1, PD-L1, PD-L2, TIM-3, MICA, MICB, Perforin, ULBP-1, ULBP-3, ULBP-4, Arginase, NT5E, Tactile, ECadherin, Nectin-2, PVR, Siglec-7, Siglec-9, B7-H6, B7-H3, IAP, BLAST-1,OX40, ICOS Ligand, TIMD-4, S100A8/A9, and VISTA.

The level of the circulating tumor biomarkers, CA-125, CA-15-3, CA-19-9, CEA, and CYFRA-21, was detected according to manufacturers’ instruction, using the customized MILLIPLEX Human Circulating Cancer Biomarker Panel 1 kit (Merck KGaA, Germany).

The concentration of serum immune checkpoint mediators and circulating tumor biomarkers was measured by Luminex Bio-Plex 200 system (BIO-RAD). Acquisition and data analysis were performed by Bio-plex Manager TM version 6.2 software. Analyte concentrations in patients were calculated against a seven-point standard curve using a five-parametric fit algorithm in xPONENT v4.0.3.

### Measurement of PD-L1 expression in tumor tissue

**Figure 1:**
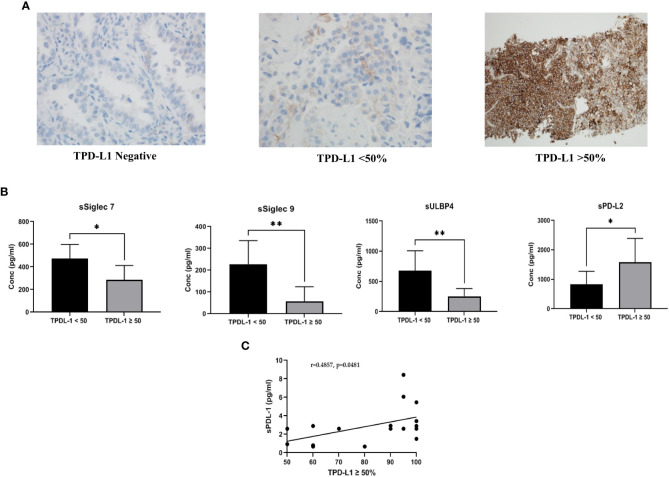
**(A)** Representative images of PD-L1 expression on tumor tissue: Tissue PD-L1 negative, tissue PD-L1<50% and tissue PD-L1>50% was observed by immunohistochemistry using DAKO PD-L1 IHC 22C3 pharmDx assay **(B)** Comparison of soluble immune biomarker expression between TPD-L1 low (<50%) and TPD-L1 high (>50%) groups: Significant down regulation of immune inhibitory checkpoint markers, sSiglec7 (p=0.011*), sSiglec9 (p=0.003**), sULBP4 (p=0.008**) and significant up regulation of sPD-L2 (p=0.015*) was observed in high TPD-L1 (>50%) expressing group **(C)** Pearson correlation showed significant moderate positive linear correlation (r =0.4857, p=0.048*) between the immune inhibitory marker, soluble PD-L1 and high TPD-L1(>50%) expressing group.

TPD-L1 expression was performed in the CAP-accredited Department of Laboratory Medicine and Pathology (DLMP), HMC, Qatar, as part of routine diagnostic testing. TPD-L1 expression was assessed, as per manufacturers’ instructions, on formalin-fixed, paraffin-embedded (FFPE) tissue, by a qualitative immunohistochemical assay (DAKO PD-L1 IHC 22C3 pharmDx) using monoclonal mouse Anti-PD-L1, Clone 22C3 on Automated Autostainer Link 48 (Dako, USA). Briefly, following incubation with the primary monoclonal antibody to TPD-L1 or the Negative Control Reagent (NCR), specimens were incubated with a Linker antibody specific to the host species of the primary antibody, and then incubated with a ready-to-use visualization reagent, consisting of secondary antibody molecules and horseradish peroxidase molecules coupled to a dextran polymer backbone. The enzymatic conversion of the subsequently added chromogen resulted in the precipitation of a visible reaction product at the site of the antigen. The entire slide was evaluated by an independent pathologist using a light microscope objective of 10-40X. To ensure run quality control, the slides were examined in the order of hematoxylin and eosin (H&E), control cell line slide, positive control tissue slides, negative control tissue, patient tissue slide stained using the NCR, and patient tissue slide stained using the PD-L1 primary antibody slides. For TPD-L1 scoring, a minimum of 100 viable tumor cells, negative and positive controls, were tested for quality control and test validity. TPD-L1 protein expression was determined by using Tumor Proportion Score (TPS), which is the percentage of viable tumor cells showing partial or complete membrane staining. The specimen was considered PD-L1 weak positive if membrane staining of TPS≥ 1% but < 50% of the viable tumor cells was observed, high PD-L1 (strongly positive) if TPS≥ 50% of the viable tumor cells exhibited membrane staining at any intensity. The intensity was evaluated as follows: No staining scored as “0”, Weak staining as “1+”, Moderate staining as “2+”, Strong staining as “3+”. The specimen was considered PD-L1 positive if ≥1% of the viable tumor cells exhibited membrane staining at any intensity (regardless of degree intensity, 1+, 2+, 3+). Representative TPD-L1 negative, TPD-L1<50% and TPD-L1>50% IHC images (400 x magnifications) are shown in **Figure 1A**.

### Next generation sequencing for determination of genetic aberrations

Next Generation Sequencing to detect genetic aberrations was performed in the CAP-accredited Department of Laboratory Medicine and Pathology (DLMP), HMC, Qatar, as part of routine diagnostic testing. The NGS Oncomine Focus Assay was performed for the samples. A total of 52 genes were tested to cover hotspots and copy number variations (CNVs) by DNA sequencing and most targeted gene fusions by RNA sequencing in a single workflow within the same NGS panel. The tumor area was collected from slides of a formalin-fixed paraffin-embedded (FFPE) specimen; this area was identified by the consultant pathologist from which genomic DNA/RNA was extracted and analyzed by using Next Generation Sequencing NGS – Ion S5 (Oncomine Focus Assay). The data generated were analyzed for alterations in the Hotspot genes and fusion drivers.

### Clinical assessment of response

Response to treatment was assessed *via* PET-CT imaging data and clinical assessment per RECIST criteria. Progression-free survival (PFS) was defined as the period from blood sample collection (before the first dose of anti-PD-1/PD-L1/Combined chemoimmunotherapy) to the date of clinical and radiological disease progression or death by any cause observed within 6-8 months from the start of the treatment.

### Statistical analysis

Statistical analysis was performed using GraphPad Prism version 9.3.2 (GraphPad Software, Inc., USA). Descriptive statistics including median (IQR), 95% CI and frequencies (%) were used for analysis of demographics and soluble biomarker concentrations. Mann-Whitney U test was used for analyzing differences in biomarkers expression levels in TPD-L1 groups, treatment response, and response in different treatment types. The correlation between TPD-L1 and soluble biomarkers was determined by Pearson correlation. Cut-off values of soluble biomarkers were estimated by receiver operating characteristic (ROC) curve. Association of cut-off values with demographic/clinical characteristics was performed by Fisher exact test. Survival curves were plotted using the Kaplan-Meier method and compared using the log-rank test. Univariate and multivariate analyses of Progression-free survival (PFS) were performed using the Cox Proportional Hazard regression model with hazards ratio (HR) and 95%CI. The results were considered statistically significant if p<0.05 was observed.

## Results

### Demographic and clinical characteristics

A total of 31 advanced-stage, metastatic NSCLC patients were enrolled in the study. The demographic and clinical characteristics of patients are shown in **Table 1**. Anti-PD-1 treatment was administered to 48% of the patients (Pembrolizumab 35%, Nivolumab 13%), while 10% of the patients were treated with anti-PD-L1 (Durvalumab). The remaining 42% of the patients were treated with combined chemoimmunotherapy *(*Pembrolizumab+Carboplatin+Pemetrexed). Response to treatment was observed in 48% (n=15) of the patients, while 52% of the patients (n=16) were categorized as non-responders (**Table 1**).

**Table 1:** Patient characteristics (all, responders and non-responders) and their association with treatment response.

Patient Characteristics	Patients n=31 (%)	Responders (R) n=15 (%)	Non-Responders (NR) n=16 (%)	Association analysis R. vs. NR (p value)
Age in years (Median, range)	59 (40-80)			
<60	16 (52)	5 (33)	11 (69)	0.756
>60	15 (48)	10 (67)	5 (31)	
Gender				
Male	26 (84)	12 (80)	14 (88)	0.6539
Female	5 (16)	3 (20)	2 (12)	
Ethnicity				
Arabs	14 (45)	8 (53)	6 (38)	0.4795
Non-Arabs	17 (55)	7 (47)	10 (62)	
Smoking history				
Never	10 (32)	6 (40)	4 (25)	0.4578
Current/Former	21 (68)	9 (60)	12 (75)	
Histology				
Adenocarcinoma	27 (87)	13 (87)	14 (88)	0.999
Squamous cell carcinoma	4 (13)	2 (13)	2 (12)	
Stages				
Stage 3	7 (23)	6 (40)	1 (6)	0.0373*
Stage 4	24 (77)	9 (60)	15 (94)	
Differentiation status				
Well differentiated	10 (32)	4 (27)	5 (31)	0.999
Poorly differentiated	21 (68)	11 (73)	11 (69)	
ECOG PS				
0-1	26 (84)	13 (87)	13 (81)	0.999
>2	5 (16)	2 (13)	3 (19)	
Genetic alterations				
EGFR				
Wild type	28 (90)	14 (100)	14 (88)	0.999
Mutated	1 (3)	–	1 (6)	
Unknown	2 (7)	–	2 (12)	
ALK				
Wild type	27 (87)	14 (93)	13 (81)	0.451
Mutated	1 (3)	1 (7)	–	
Unknown	3 (10)	–	3 (19)	
ERBB3				
Wild type	28 (90)	14 (93)	14 (88)	0.999
Mutated	3 (10)	1 (7)	2 (12)	
*KRAS*				
Wild type	28 (90)	13 (87)	15 (94)	0.5996
Mutated	3 (10)	2 (13)	1 (6)	
PDL-1 TPS				
Negative	8 (26)	1 (7)	7 (44)	
*TPD-L1 Positive < 50%*	6 (26)	3 (21)	3 (33)	
*TPD-L1Positive >50%*	17 (74)	11 (79)	6 (66)	0.6430
Brain metastasis	17 (74)			
Present	15 (48)	8 (53)	7 (44)	0.7244
Absent	16 (52)	7 (47)	9 (56)	
Liver Metastasis				
Present	7 (23)	1 (7)	6 (38)	0.0829
Absent	24 (77)	14 (93)	10 (62)	
Pulmonary Metastasis				
Present	21 (68)	8 (53)	13 (81)	0.1351
Absent	10 (32)	7 (47)	3 (19)	
Previous history of radiotherapy				
Yes	14 (45)	9 (60%)	8 (50)	0.7224
No	17 (55)	6 (40%)	8 (50)	
Previous lines of chemotherapy				
0	8 (26)	5 (33%)	3 (19)	0.4331
>1	23 (74)	10 (66%)	13 (81)	
Treatment type				
Anti-PD-1 (Pembrolizumab/Nivolumab)	15	6 (40)	9 (56)	0.2059
Anti-PD-L1(Durvalumab)	3 (10)	3 (20)	0 (0)	
Chemoimmunotherapy (Pembrolizumab+Carboplatin+Pemetrexed)	13 (42)	6 (40)	7 (44)	0.999 (anti-PD-1) 0.2125 (anti-PD-L1)

ECOG PS, Eastern Cooperative Oncology Group performance status; EGFR, Epidermal Growth factor receptor; ALK, Anaplastic lymphoma kinase; ERBB3, Erb-b2 receptor tyrosine kinase 3; KRAS, Kirsten rat sarcoma viral oncogene homolog; PD-L1, programmed death-ligand 1; PD-1, Programmed cell death Protein 1; TPS, Tumor Proportion score.

### Expression of soluble immune checkpoints/circulating tumor antigens and patients’ characteristics

The concentration of soluble immune checkpoints/circulating tumor antigens was successfully detected, and median + Interquartile (IQR) values of tested biomarkers are shown in **Supplementary table 1**.

### Expression of TPD-L1 in enrolled patients

TPD-L1 expression was observed in 74% (n=23), while 26% (n=8) of the patients were found to be negative. For further analysis, TPD-L1 positive patients were stratified into two groups: TPD-L1<50% (n=6) and TPD-L1>50% (n=17). Representative images for TPD-L1 negative, TPD-L1<50% and TPD-L1>50% are shown in **Figure 1A**.

### Soluble biomarkers and TPD-L1

Comparison of the expression level of soluble biomarkers between TPD-L1 negative vs. positive groups showed no significant change. However, comparison of TPD-L1<50% and >50% groups showed significant changes in various soluble markers. In the TPD-L1>50% group, significant downregulation of the immune inhibitory checkpoint markers, sSiglec7 (p=0.011*), sSiglec9 (p=0.003**), sULBP4 (p=0.008**) and significant up-regulation of sPD-L2 (p=0.015*) was observed (**Figure 1B**). The result indicates that high TPD-L1 expression could induce secretion of the soluble Natural Killer (NK) and T cell immune inhibitory checkpoint markers for immune regulation of anti-tumor response. The median (IQR) values of soluble biomarkers in TPDL-1<50% and TPD-L1>50% groups are given in **supplementary Table 2**.

### Correlation between soluble immune checkpoint biomarkers and TPD-L1 >50% group

Pearson correlation analysis was performed to understand the linear relationship of TPD-L1 expression with up/down regulated soluble markers sSiglec7, sSiglec9, sULBP4, and sPD-L2. In addition to these markers, correlation analysis between TPD-L1 and sPD-L1 was also performed to determine if there is an existing relationship between the tissue and the secreted form of PD L1. No significant correlation between TPD-L1 >50% group and sSiglec7, sSiglec9, sULBP4, sPDL2 was noted. However, a moderate positive linear correlation (r =0.4857) was observed between the immune inhibitory marker, sPD-L1, and TPD-L1 >50%, with a significance value of p=0.048* (**Figure 1C**). This indicates that TPD-L1 expression levels are directly proportional to the concentration of sPD-L1 i.e., as TPD-L1 expression increases above 50%, the concentration of sPD-L1 also increases, making sPD-L1 a potential surrogate marker for longitudinal monitoring of TPD-L1.

### Expression of soluble biomarkers in TPD-L1 >50% group and their role in treatment response

**Figure 2:**
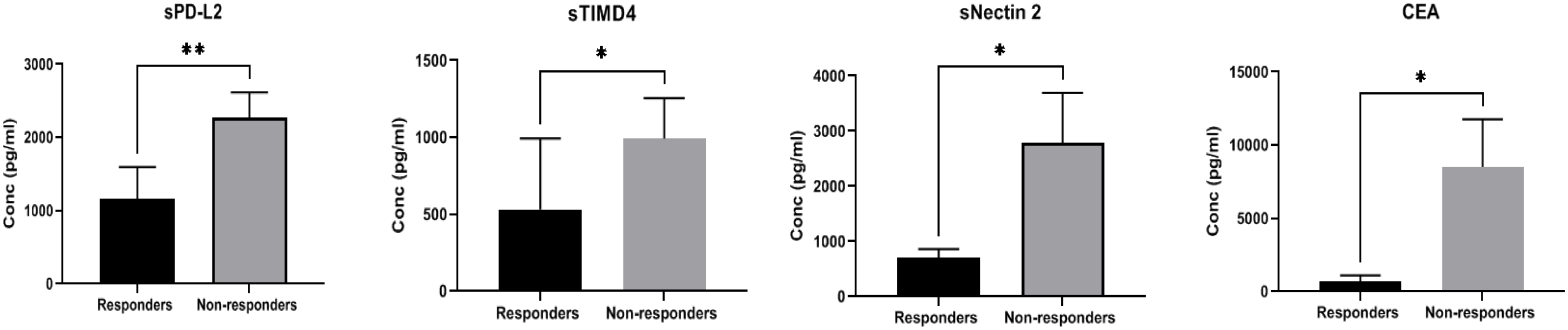
Comparison of soluble biomarker expression between responders (n=6) and non-responders (n=11) in high TPD-L1(>50%) group showed significant down regulation of immune inhibitory markers sPD-L2 (p=0.008**), sTIMD4 (p=0.040*), sNectin2 (p=0.012*) and CEA (p=0.024*) in responding patients.

A comparison of the expression of soluble biomarkers with treatment response was performed in TPD-L1 groups. In TPD-L1 >50% group, comparison between responders (n=6) and non-responders (n=11) showed significant down regulation of immune inhibitory markers sPD-L2 (p=0.008**), sTIMD4 (p=0.040*), sNectin2 (p=0.012*) and CEA (p=0.024*) in responding patients (**Figure 2**). Our study results imply that in patients expressing TPD-L1 >50%, T cell immune checkpoint and circulating tumor antigens may play a role in immune modulation and tumor response. As such, these biomarkers may have utility as predictive biomarkers of response in this cohort. No significant expression of soluble biomarkers with treatment response was observed in TPD-L1 positive/negative groups and TPD-L1 <50% group (data not shown). The median (IQR) values of soluble biomarkers in responders vs. non-responders in the TPD-L1>50% group is given in **Supplementary Table 2**.

### Association of patient characteristics with treatment response

Based on imaging and clinical status as per RECIST criteria, the enrolled participants were stratified as responders (n=15) and non-responders (n=16). Association of treatment response with demographic/clinical characteristics showed significant association of disease stage 4 (p=0.037*) with non-responders. No other demographics/clinical characteristics were associated with treatment response (**Table 1**).

### Soluble biomarkers and their association with treatment response in anti-PD-1/PD-L1 monotherapy and chemo-immunotherapy group

Treatment types utilized for patients included monotherapy with anti-PD1 (Nivolumab, Pembrolizumab), anti-PD-L1 (Durvalumab) and combination chemoimmunotherapy (Carboplatin +Pemetrexed+ Pembrolizumab). Due to the different treatment types, we stratified the patients into two groups. Group 1 comprised all patients who received anti-PD-1 and anti-PD-L1 monotherapy (anti-PD-1/PD-L1 monotherapy group: Nivolumab+Pembrolizumab+Durvalumab: n=18), whereas Group 2 included all patients who received combination chemoimmunotherapy (n=13).

**Figure 3:**
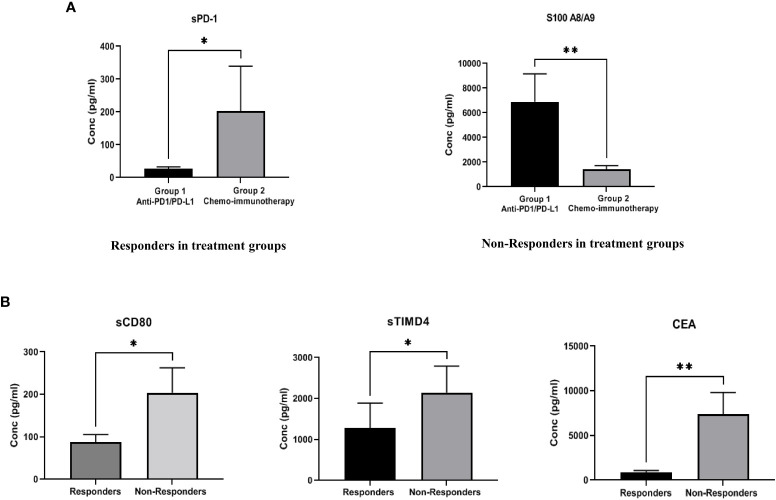
**(A)** Comparison of soluble biomarker expression between responders and non-responders in two treatment groups-Group 1 (anti-PD-1/PD-L1 monotherapy group), Group 2 (combination chemoimmunotherapy group). In “responding” patients, the immune inhibitory checkpoint marker sPD-1, was significantly down regulated (p=0.012*) in Group 1 as compared to Group 2. In “non-responding” patients, the immune suppressive biomarker S100A8/A9 (p=0.0084**) was significantly up regulated in Group 1 as compared to Group 2 **(B)** Comparison of soluble biomarker expression between all responders vs. all non-responders irrespective of treatment type. Significant down regulation of the immune inhibitory biomarkers sCD80 (p=0.023*), sTIMD4 (p=0.033*) and CEA (p=0.008**) in “responding” patients was observed.

The expression of soluble biomarkers was analyzed as follows a) responding patients in Group 1 (n=9) vs. Group 2 (n=6) and b) non-responding patients in Group 1 (n=9) vs. Group 2 (n=7). Interesting results were observed with both groups’ significant up/down-regulation of soluble biomarkers. In “responding” patients, the immune inhibitory checkpoint marker sPD-1, was significantly downregulated (p=0.012*) in Group 1 compared to Group 2. On the other hand, in “non-responding” patients, the immune suppressive biomarker S100A8/A9 (p=0.0084**) was significantly upregulated in Group 1 compared to Group 2. Our results clearly identify soluble biomarkers that can discriminate treatment response in different treatment groups and thus serve as predictive biomarkers (**Figure 3A**). Median (IQR) values of soluble biomarkers in responding and non-responding patients in Group 1 and Group 2 is given in **Supplementary Table 3**.

### Comparison of soluble biomarkers in responders and non-responders irrespective of treatment types

To identify generalized biomarkers of response in NSCLC patients treated with ICI, we compared the expression of soluble biomarkers in responders (n=15) vs. non-responders (n=16), irrespective of treatment groups. The results showed significant downregulation of the immune inhibitory biomarkers sCD80 (p=0.023*), sTIMD4 (p=0.033*), and CEA (p=0.008**) in “responding” patients indicating that these biomarkers may be playing a rather generalized but extensive role in immune modulation and treatment response to ICI therapy (**Figure 3B**). The median (IQR) values of soluble biomarkers between responders and non-responders, irrespective of treatment types, is given in **supplementary Table 4**.

### Determination of optimal cut-off values of soluble biomarkers to discriminate responders from non-responders

**Figure 4:**
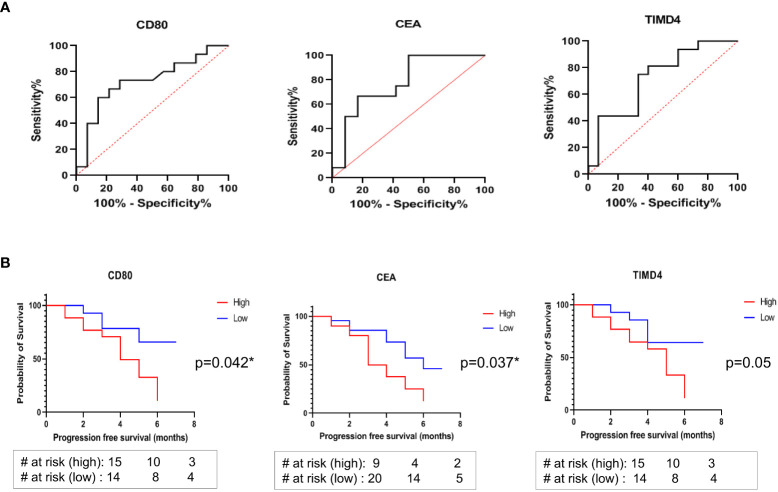
**(A)** ROC curves to discriminate responders from non-responders identified optimal cut-off values of soluble biomarkers: CD80 <91.7pg/ml, AUC: 0.7262, sensitivity: 73%, specificity: 71%; TIMD4 <600pg/ml, AUC: 0.7250, sensitivity: 75%, specificity: 66%; CEA <1614pg/ml AUC: 0.778, sensitivity: 67%, specificity: 83% **(B)** Kaplan Meier (log rank) analysis for association of cut-off values with progression free survival showed that patients having CD80 cut-off value of lower than 91.7 pg/ml (HR: 2.873, 95% CI: 1.078-7.658, p=0.042*) and CEA cut-off value of lower than 1614 pg/ml (HR: 2.566, 95% CI: 0.131-1.160, p=0.037*) were significantly associated with better progression free survival. No significant association of TIMD4 cut-off value with PFS was observed (HR: 2.699, 95% CI: 1.012-7.202, p=0.05).

The generalized soluble biomarkers that showed significant association with treatment response (irrespective of treatment types), including CD80, TIMD4, and CEA, were further analyzed by Receiver Operator Characteristic Curve (ROC) to determine their optimal cut-offs. It was found that the optimal cut-off value for soluble biomarkers to discriminate responders from non-responders were as follows: CD80 <91.7pg/ml (AUC: 0.7262, 95% CI: 0.535-0.917, sensitivity: 73%, specificity: 71%); TIMD4 <600pg/ml (AUC: 0.7250, 95% CI: 0.543 to 0.907, sensitivity: 75%, specificity: 66%); CEA <1614pg/ml (AUC: 0.778, 95% CI: 0.586-0.969, sensitivity: 67%, specificity: 83%) (**Figure 4A**). The cut-off values were further analyzed for their association with PFS in patients.

### Association of soluble immune checkpoint/circulating tumor antigens with progression free survival

The association of higher than cut-off and lower than cut-off values of the soluble biomarkers CD80, TIMD4, and CEA with PFS was determined using Kaplan Meier (log-rank) test. It was observed that patients having higher than cut-off values of CD80 and CEA had poor PFS (median survival of 4 months and 3.5 months, respectively). On the other hand, patients having CD80 cut-off value of lower than 91.7 pg/ml (HR: 2.873, 95% CI: 1.078-7.658, p=0.042*) and CEA cut-off value of lower than 1614 pg/ml (HR: 2.566, 95% CI: 0.131-1.160, p=0.037*) were significantly associated with better progression-free survival (**Figure 4B**). No significant association of TIMD4 cut-off value with PFS was observed (HR: 2.699, 95% CI: 1.012-7.202, p=0.05) (**Figure 4B**).

### Cox proportional hazard regression analysis

To assess the impact of patient characteristics and soluble biomarkers as independent predictive factors of PFS, univariate and multivariate analysis by Cox Proportional Hazard Regression was performed. Multivariate analysis showed that age <60 years (HR 4.856 [95% CI: 1.244-23.10]; p=0.031) and CEA lower than the cut-off value of 1614 pg/ml (HR 0.1834 [95% CI: 0.04-0.65]; p=0.012) are independent predictors of better progression-free survival in patients (**Table 2**).

**Table 2:** Uni- and multivariate analysis of Progression free survival by Cox proportional Hazards model.

	Univariate analysis		Multivariate analysis	
Variables	HR (95% CI)	p value	HR (95% CI)	p value
Age (>60 vs.<60)	3.034 (1.094-9.691)	0.041*	4.856 (1.244-23.10)	0.031*
PDL-1 TPS (Positive vs. Negative)	2.891 (1.024-7.849)	0.037*	2.019 (0.679-5.728)	0.188
Liver Metastasis (Absent vs. Present)	4.199 (1.310-13.38)	0.013*	2.351 (0.607-8.938)	0.204
CEA (High vs. Low)	0.357 (0.129-0.984)	0.042*	0.183 (0.04-0.65)	0.012*

## Discussion

We have identified in this study immune inhibitory/stimulatory soluble mediators as a potential surrogate/predictive biomarker for TPD-L1 status, treatment response, and progression-free survival in NSCLC patients treated with anti-PD-1/PD-L1. This a pilot study and the results showed a significant association of circulating tumor antigen, CEA, and several NK and T cell immune checkpoint markers with TPD-L1 expression and treatment response. To the best of our knowledge, this is the first study that extensively examines the role of NK/T cell immune checkpoint biomarkers/circulating tumor antigens with regards to TPD-L1 expression and treatment response in this cohort of patients.

We first aimed to identify and understand the role of various NK and T cell immune checkpoint serum markers as surrogate biomarkers/predictors of response with respect to TPD-L1 status. TPD-L1 is the only FDA approved companion diagnostic, predictive marker to assess the eligibility of NSCLC patients for ICI treatment (42). The ICI treatments for NSCLC include anti-PD-1, anti-PD-L1, or combined chemoimmunotherapy. Although TPD-L1 assessment is not a pre-requisite for all ICI treatments, several clinical trials have evaluated its role in predicting survival benefits for ICI-treated NSCLC patients (43). A large-scale meta-analysis on fifteen randomized controlled trials showed that patients with high TPD-L1 expression (>50%) exhibited improved overall response rates and subsequently benefitted from anti-PD-1/PD-L1 therapy (33). However, TPD-L1 expression could not predict survival benefits in patients on combined chemoimmunotherapy (33). This variability in predicting immunotherapy efficacy is possibly due to its inherent limitations, including inadequate tissue sampling, tumor heterogeneity, variable testing parameters, and evolutionary changes in TPD-L1 expression (induced by prior treatment lines), making its utility in clinical settings unclear. On the other hand, liquid biopsy, with its fundamental characteristics, such as noninvasiveness, incorporating tumor heterogeneity, ease of longitudinal monitoring *via* multiple sampling, and representation of systemic biomarker expression, could serve as an essential component to assess immunotherapy efficacy (44). Furthermore, its utility as a surrogate marker for TPD-L1 expression can help in longitudinal treatment monitoring. Our results showed that in patients with TPD-L1 >50% expression, significant downregulation of the soluble NK immune inhibitory markers Siglec-7 and-9, ULBP4 and significant upregulation of the soluble T cell immune inhibitory marker PD-L2 was observed. The role of these markers in immune regulation is well documented. Siglecs (Sialic acid-binding immunoglobulin-like lectins) are a family of receptors, present mainly on immune cells (45),. Siglec receptors recognize sialoglycan ligands on cell membranes and lead to eventual dephosphorylation of downstream immune pathways leading to inhibition of cellular activation (45). In tumors, the immune suppressive microenvironment helps facilitate this inhibition *via* aberrant expression of sialoglycan ligands on tumor cells and Siglec receptor overexpression on immune cells (46, 47). A strong receptor-ligand binding leads to immune inhibition and tumor escape (46, 47). Studies have shown that Siglec-7 and -9 are abundantly present in NK cells, and their interaction with sialoglycan ligands (on tumor cells) inhibits NK cell activation (48). Enhanced expression of siglec-7 and -9 in peripheral CD8^+^ T cells and tumor tissues have been observed in NSCLC, melanoma, and colon cancers (49, 50). Moreover, a study on NSCLC patients observed that high Siglec-9 expression on infiltrating CD8^+^ T cells was associated with increased expression of PD-L1, co-expression of inhibitory receptors PD-1, TIM-3, Lag3, and reduced production of inflammatory cytokines leading to an exhausted T cell phenotype and poor survival in patients (50–52). In lieu of this, our results show a different pattern. Serum-derived Siglec-7 and -9 were downregulated in patients exhibiting TPD-L1 >50% expression. Since we could not determine the expression of Siglecs in the tumor tissue, it is possible that Siglecs were overexpressed within the tumor tissue, subsequently leading to high PD-L1 expression. However, with their release into the circulation as soluble forms, other factors within the TME may have come into play for their downregulation and modulation. Down-regulation of Siglecs has been associated with augmentation of anti-tumor responses. In this, studies in mice deficient in Siglecs-E (the functional equivalent of human Siglec-9) showed increased *in vivo* killing of tumor cells and enhanced immunosurveillance (53). The same study showed that polymorphisms in human Siglec-9 contributed to its reduced binding to cancer cells, leading to improved survival in NSCLC patients (53). Therefore, we postulate that downregulation of soluble Siglecs in circulation in our cohort may indicate their role in the anti-tumor response. However, since no study on serum Siglecs and TPD-L1 has been reported, we could not corroborate our data with previous studies. Larger studies on this aspect could provide a better understanding of these Siglecs in TPD-L1 expression and immune regulation.

Another marker, UL16-binding protein 4 (ULBP4) was found to be significantly down regulated in patients expressing TPD-L1>50%. Mainly, NK cell−mediated cytotoxicity is regulated *via* the binding of NK group 2 member D (NKG2D) activating receptors with their ligands, such as the ULBP family (ULBP1-6) (54, 55). ULBP ligand expression is observed to be low in non-malignant cells (56, 57). However, in tumors, ULBP 1-6 ligands are aberrantly expressed, leading to modulation of anti-tumor responses (56, 57). Specifically, secreted forms of ULBP4 (generated *via* alternative splicing) have been reported to bind to NKG2D receptor, thus initiating its internalization for NK cell-acquired dysfunction and reduced NK cytotoxicity for tumor immune escape (58–60). Moreover, studies have reported that as ULBP4 ligand secretion increases, it induces the expansion of immune suppressive T cells, thus creating a favorable environment for tumor growth (61). On the other hand, studies on glioma and nasopharyngeal carcinoma have documented contrasting results, showing that upregulation of the cytokines TGF-β/IFN-γ and increased PD-L1 expression can lead to selective downregulation of ULBP3 and 4 to facilitate tumor escape (62–64). Our results agree with this notion showing that as PD-L1 expression increases, ULBP4 expression decreases, possibly playing its role in immune modulation. However, since the role of soluble ULBP4 with respect to PD-L1 expression in ICI-treated NSCLC patients has not been reported yet, we believe that our results could allow further studies to explore this aspect in detail.

In addition to NK markers, the T cell immune inhibitory checkpoint ligand PD-L2 was found to be upregulated in the TPD-L1 >50% group. PD-L2 that serves as second ligand for PD-1 and is involved in T cell regulation *via* decreased cytokine production and inhibition of T cell receptor (TCR)-mediated proliferation (65). Studies on lung and melanoma have shown that simultaneous expression of PD-L1 with PD-L2 is an important concept and could be one of the mechanisms utilized by tumor cells for immune evasion and tumor progression (66, 67). In fact, a study on ovarian cancer reported that blocking both PD-L1 and PD-L2 could help to overcome resistance to ICI treatment by unleashing the immune responses, thus indicating a clear role of both ligands in immune regulation (68). In our study, we observed simultaneous upregulation of sPD-L2 with TPD-L1 expression, indicating a possible synergistic effect for tumor response. Though tissue PD-L2 was not tested in our cohort, we assume that soluble PD-L2 (generated *via* splicing event of membrane-bound PD-L2) may indicate its presence within the tumor tissue. Also, as our result indicates concurrent up regulation of both markers (PD-L1 and PD-L2), we propose the utility of sPD-L2 as a surrogate marker for tissue PD-L1 and PD-L2. However, since limited studies on sPD-L2 are available in the literature, our assumption on the dualistic role of TPD-L1 and soluble PD-L2 in anti-tumor response needs further validation.

To understand if any linear relation exists between the up/down regulated soluble markers Siglec-7,-9, ULBP4 and PD-L2, Pearson correlation analysis was performed. We did not find any of these markers to correlate with TPD-L1. However, we did correlation analysis of sPD-L1 with TPD-L1 with the concept that since sPD-L1 is a spliced variant secreted by membrane-bound PD-L1, a linear relationship could exist between the two markers. Interestingly, correlation analysis between serum PD-L1 and TPD-L1 >50% showed a moderate positive relationship indicating that increased serum concentration of PD-L1 could be associated with increased PD-L1 expression in tissues. This is an important finding and allows the assumption that serum PD-L1 could be utilized as a surrogate marker for TPD-L1 status for longitudinal monitoring in patients on ICI treatment. Studies showing a significant positive correlation between the two markers have been reported, thus corroborating our observation (69, 70).

Furthermore, we aimed to identify specific biomarkers that could help stratify responding from non-responding patients in TPD-L1 >50% group. This is important as the identification of early biomarkers of response could help treatment management in this group. In responding patients with TPD-L1 >50% expression, the immune inhibitory markers sPD-L2, sTIMD4, sNectin2 and CEA were significantly downregulated. sPD-L2 is a spliced variant of membrane-bound PD-L2 that retains the ability to bind to its membrane-bound PD-1 receptor for immune regulation (71). Studies on the prognostic value of sPD-L2 in NSCLC are very limited. Only one study on 22 patients was carried out that evidenced better survival in patients with low pre-treatment sPD-L2 expression (18, 72). Moreover, co-expression of sPD-L2 with other soluble mediators such as PD-L1, CD137, TIM-3 BTLA-4 and CEA has been associated with favorable clinical response indicating a synergistic effect of these soluble mediators with each other to induce modulatory effects within the tumor microenvironment (18, 72). In our study, we observed downregulation of sPD-L2 with other soluble immune inhibitory markers such as sTIMD4, sNectin2, and CEA indicating the plausibility of a synergistic mechanism of soluble markers with each other thus enabling anti-tumor response in high tissue PD-L1 expressing patients. Further studies on these markers would enable a better understanding on this inference.

Besides sPD-L2, the NK associated ligand, sNectin2 was also found to be down regulated in high tissue PD-L1 responding patients. Nectin-2 is a immunoglobulin-like cell-to-cell adhesion protein that acts in a stimulatory or inhibitory manner Several studies on serum Nectin-2 have associated its overexpression with aggressiveness and metastasis in various cancers including colon, breast, esophageal and lung indicating its role as a prognostic and predictive biomarker in cancers (73–76). Moreover, blockade *via* anti-Nectin-2 monoclonal antibodies can induce antibody-dependent cellular cytotoxicity (ADCC) with robust anti-tumor response in breast and ovarian cancers, indicating its role in immune regulation (77, 78). Similar results were observed for Esophageal squamous cell carcinoma (ESCC) where knockdown of Nectin−2 in ESCC cell lines was associated with effective suppression of cell migration and invasion (75). Our results corroborate with these studies, and we postulate that high TPD-L1 could lead to immune-inflamed TME with downregulation of sNectin-2 as an anti-tumor response mechanism in responding patients of this cohort.

Our results also showed downregulation of the immune inhibitory marker TIMD4 (T Cell Immunoglobulin and Mucin Domain Containing 4) in TPD-L1 >50% group. TIMD4 is a cell-surface glycoprotein and in cancers including renal cell carcinoma, diffuse large B-cell lymphoma, pancreatic cancer, and glioma, expression of TIMD4 has been associated with enhanced apoptosis, reduced clonogenic ability of cancer cells, and better survival (79–82). In NSCLC, a comprehensive study documented the role of TIMD4 overexpression in the promotion of lung cancer cell proliferation and poor overall survival (83). Although the mechanism of TIMD4-mediated cancer progression remains unknown, the study showed that mutation in the TIMD4 RGD motif reduces cancer progression (83). We presuppose here (based on the mechanism of action of TIMD4) that high PD-L1 expression could have influenced the TME to induce downregulation of circulating TIMD4 as an active anti-tumor response mechanism in responding patients.

In addition to T and NK cell markers, we also found circulating tumor antigen CEA to be downregulated in the high TPD-L1 group. CEA is a serum glycoprotein and is a well-established prognostic and predictive tumor marker utilized for treatment monitoring in various cancers (84–86). In lung cancers, elevated CEA levels have been associated with tumor size, lymph node status, stage of disease, and treatment monitoring (87). Studies on ICI-treated NSCLC patients’ have associated high pre-treatment levels of CEA with worse PFS and OS (23, 25, 27). Moreover, a study on the correlation between CEA and PD-L1 has reported CEA as an independent prognostic indicator of worse OS in the PD-L1-positive group (88). On the other hand, a more specific role of CEA and immune modulation *via* PD-L1 has recently been documented (89–94). Several studies on T cell–bispecific antibody (CEA-TCB) targeting CEA and T cell receptor have shown interesting results in syngeneic tumor models, cell lines, *in vivo* humanized mice, and patients (89–94). CEA-TCB specifically induced T cell-mediated killing of CEA-expressing tumors by converting a non-inflamed PD-L1 negative tumor to a highly inflamed PD-L1 positive tumor (89–94). In our study, responding patients with high tissue PD-L1 showed down-regulation of CEA. Based on previous studies discussed above including low pre-treatment CEA associated with response and elevated PD-L1 expression inducing an immune hot/inflamed TME, we postulate that in our cohort high PD-L1 expression may have led to downregulation of CEA thus facilitating an efficient anti-tumor response.

The second aim of our study was to understand the role of soluble biomarkers as early predictors of response in NSCLC patients on ICI treatment. We stratified our analysis into various aspects, as discussed below. Firstly, we sought to identify early predictive biomarkers of response in patients on different therapeutic regimens (anti-PD-1/PD-L1 monotherapy group vs. chemoimmunotherapy group). In the anti-PD-1/PD-L1 monotherapy group, we identified two immune suppressive markers to be significantly associated with response. In responding patients, immune inhibitory checkpoint marker sPD-1 was found to be significantly downregulated. sPD-1 is a spliced variant of membranous PD-1 that retains its PD-L1 binding domain and can thus bind to membranous PD-L1 and PD-L2. This binding facilitates several immune modulatory effects, including early activation of CD8^+^ T cells, blocking of PD-L1 expression on tumor cells, and essentially reducing T cell inhibition (11, 95). On the other hand, some studies have documented its role in tumor immune escape *via* its ability to bind with membrane-bound PD-1 and in turn, compete with therapeutic anti-PD-1 monoclonal antibodies for their PD-1 binding site (95). The successful binding of sPD-1, instead of anti-PD-1 antibodies, leads to suboptimal efficacy/reduced bioavailability of therapeutic monoclonal antibodies (95). In ICI- treated NSCLC patients, the role of sPD-1 is still unclear and is described in a dynamic context (18, 19, 96). Mainly, dynamic increase in sPD-1 after anti-PD-1 treatment has been significantly associated with disease progression, indicating that as sPD-1 levels increase, it strengthens T cell inhibition and cancer immune evasion, thus resulting in poor outcome (18, 19, 96). Our result shows that in the anti-PD-1/PD-L1 group, low pre-treatment sPD-1 levels are associated with patients’ response to treatment. We postulate that low expression of sPD-1 may induce a weak affinity for membranous PD-1 thus allowing benefit to therapeutic anti-PD-1 antibodies to effectively bind and induce an active anti-tumor response. However, since we did not assess its modulation after treatment, we cannot comment on its dynamic role in immune regulation (as described in earlier studies). Our group is conducting a study on pre- and post-treatment sPD-1 levels which may give better insight into this aspect.

We also identified S100A8/A9 as a biomarker in non-responding patients on anti-PD-1/PD-L1 monotherapy. In tumors, pro-inflammatory S100A8/A9 production helps sustain MDSC accumulation for maintaining immune suppressive TME and facilitating tumor immune escape (97, 98). In lung cancers, S100A8/A9 overexpression has been implicated in the promotion of pre-metastatic niches, anchorage-independent invasion, and tumor cell proliferation (99, 100). Several studies on NSCLC have also associated overexpression of S100A8/A9 with poor survival and a high relapse rate (100–103). Moreover, the blockade of S100A8/A9 by anti-S100A8/A9 monoclonal antibodies demonstrated significant inhibition of lung metastasis in a mouse model (104). With respect to anti-PD-1 treatment, studies on head and neck, gastric, and melanoma have reported high levels of S100A8/A9 in non-responding patients indicating its role in ICI treatment resistance (105–108). However, studies on the role of S100A8/A9 in NSCLC patients treated with immune checkpoint inhibitors are limited. One single study, conducted on extracellular vesicle (EVs) proteins in 31 ICI-treated NSCLC patients, reported dynamic modulation of S100A8 with increased baseline associated with increased chemotaxis of myeloid cells (S100A8) while decreased expression (after treatment) was associated with inhibition of myeloid cell chemotaxis with induction of treatment response (109). Our result supports such a mechanism where the increased expression of S100A8/A9 may lead to increased chemotaxis of myeloid cells, and this resulted in immune suppression and resistance to the response. Additionally, results from other cancers (described above) corroborate with our study findings indicating the significance of S100A8/A9 as a novel predictive biomarker in ICI-treated NSCLC patients.

Having identified discriminatory markers in different treatment types, we intended to evaluate the predictive biomarkers of response irrespective of the treatment types used. This objective aimed to identify generalized biomarkers that could help to stratify responders vs. non-responders in patients on any type of ICI regimen. We observed downregulation of sCD80, CEA and sTIMD4 in responding patients. For TIMD4, the optimal value of <600 pg/ml was found to discriminate responders from non-responders with sensitivity and specificity of 75 to 66%, respectively. However, this optimal value could not be associated significantly with PFS. As discussed earlier, the mechanism of TIMD4 is still unclear. However, its low expression has been associated with better overall survival in NSCLC, indicating its potential as a prognostic/predictive biomarker (81, 83, 110). Since our results did not show its association with the response (as observed in previous studies), we hypothesize that synergistic expression of circulating immune modulatory molecules such as CD80, CEA, etc., with TIMD4 may be playing their role in influencing its association. Furthermore, it is possible that the role of TIMD4 as a predictive biomarker may be associated with its dynamic modulation in pre- and post-treatment samples.

In addition to sTIMD4, an optimal cut-off value of sCD80 level (<91.7 pg/ml) was found to be able to discriminate responders from non-responders and PFS. Briefly, soluble CD80 is generated *via* splicing of membranous CD80 (111). Though sCD80 lacks a transmembrane domain, it can still bind to CTLA-4, CD28 and activated T cells (111). Based on its ability to interact with both co-stimulatory (CD28) and co-inhibitory (CTLA-4) molecules, its role in immune modulation is contradictory. Its engagement with CD28 and PD-L1 is associated with T cell activation, while it’s binding with CTLA-4 can lead to co-inhibition of T cells leading to tumor immune escape and progression (112, 113). Moreover, sCD80 can compete with membrane-bound mCD80 on antigen-presenting cells thus reducing its co-stimulatory effects on T cells making the tumor invisible to the immune cells (114). Studies on prostate cancer, hematological malignancies, renal cell carcinoma, and NSCLC have associated low serum CD80 expression with progression-free survival while high levels are associated with enhanced invasiveness and poor prognosis (115–118). In this context, our results corroborate with previous findings. However, in our study, multivariate analysis did not identify sCD80 as an independent predictive biomarker in this cohort. This could be due to the inherent characteristic of this marker to form intricate, complex relationships with other checkpoints such as PD-L1, CD28, and CTLA4, making it a dynamic rather than an independent marker (119, 120). Larger comprehensive studies on sCD80 will help to provide a better understanding of this marker in ICI- treated NSCLC patients.

Our study identified CEA as a highly robust predictive biomarker in the ICI-treated NSCLC patient cohort. The optimal cut-off value of CEA <1614 pg/ml was associated with not only its ability to discriminate responders vs. non-responders but also with PFS and as an independent predictor of response. The role of CEA in its prognostic/predictive capacity has been documented for several cancers (23, 25, 27). However, limited studies have reported on this important tumor marker in ICI-treated NSCLC patients. Results from these studies showed high baseline CEA levels followed by a decrease of more than or equal to 20% within 4-6 weeks of immunotherapy treatment to be associated with response (23, 25, 27). Our study is the first to associate a specific cut-off, observed prior to treatment, to be associated with response prediction. As CEA is a routinely used marker in diagnostic settings, its utility in ICI treatment is complemented by this cut-off-value that could help in the early stratification of patients for efficient treatment management. Moreover, the mechanism of CEA in immune modulation (discussed earlier) further evidences its potential as a robust predictive biomarker in NSCLC patients treated with ICI.

The main limitation of this study is that we were unable to evaluate serum levels of immunosuppressive factors in a control group of individuals without NSCLC with approximately the same age and comorbidity profile as the patients. Since comorbidities such as atherosclerosis, inflammatory diseases, metabolic disorders, lifestyle and age are important factors of immune landscape change and can significantly influence the level of immunosuppressive mediators and cells in the blood, this could give a broader understanding of the immune mediators. However, due to the scope of study focusing only on patients and non-availability of healthy controls of same age and comorbidity profile as the patients, we were unable to assess this aspect.

## Conclusions

Identifying soluble, non-invasive immune oncology and tumor antigens as biomarkers of response in ICI treated-NSCLC cohort is an emerging and exciting field that can help better understand immune regulatory mechanisms and their role in anti-tumor responses. This understanding can help to stratify responding patients from non-responding ones early in the treatment timeline thus aiding in robust treatment management. We were able to identify NK/T cell markers as biomarkers for TPD-L1 and CEA as robust predictive biomarkers of response in the ICI-treated NSCLC patient cohort. We have presented several novel early biomarkers concerning TPD-L1 expression and treatment response that have not been reported in previous studies, which is the main strength of this study. However, limitations of the study include a small sample size in a single-center study. We tried to overcome these limitations with robust analysis with recommendation that our study results serve as a foundation for large-scale studies for better patient stratification and management.

## Data availability statement

The original contributions presented in the study are included in the article/**Supplementary Material**. Further inquiries can be directed to the corresponding author.

## Ethics statement

The studies involving human participants were reviewed and approved by The Institutional Review Board, Medical Research Center, Hamad Medical Corporation, Doha, Qatar. The patients/participants provided their written informed consent to participate in this study.

## Author contributions

Conceptualization, supervision, validation: AR, RM, AK, SD. Data curation, formal analysis, Software: AR, MI. Funding acquisition: AR, SD. Investigation, methodology, resources: AR, SA, AZ, SH, KP, AA-S, IA-B, WA, RA-A, SAS, SH. Project administration: AR, AP, SV, SH, MT. Visualization, roles/writing - original draft: AR. Writing - review & editing: AR, MM, VP, SU, SD, UA. All authors contributed to the article and approved the submitted version.

## Glossary

**Table:**

ADCC	Antibody-Dependent Cellular Cytotoxicity
ALK	Anaplastic Lymphoma Kinase;CA-125, Carbohydrate Antigen 125
CD80	Cluster of Differentiation 80;CEA, Carcinoembryonic Antigen
CYFRA21-1	Cytokeratin Fragment 19
DC	Dendritic cells;DNAM-1, DNAX accessory molecule 1;ECOG PS, Eastern Cooperative Oncology Group performance status
EGFR	Epidermal Growth Factor Receptor;ERBB3, Erb-b2 receptor tyrosine kinase 3;ICIs, Immune Checkpoint Inhibitors
IFN-γ	Interferon-Gamma
IHC	Immunohistochemistry
IL-2	Interleukin-2
ITIM	Immunoreceptor Tyrosine-based Inhibition Motif
KRAS	Kirsten rat sarcoma viral oncogene homolog
MDSCs	Myeloid Derived Suppressor Cells
Nectin2	Poliovirus receptor-related 2
NK	Natural killer
NKG2DL	Natural Killer Group 2D Receptor and Ligands
NSCLC	Non-small cell lung cancer
ORR	Objective Response Rate
OS	Overall Survival
PD-1	Programmed Death Protein 1
PD-L1	Programmed Death Ligand 1
PD-L2	Programmed Death Ligand 2
PFS	Progression Free Survival
PVRIG	PVR-related Ig domain
RECIST	Response Evaluation Criteria In Solid Tumors
RGD	Arginine-Glycine-Aspartic acid
ROC	Receiver Operator characteristic curve
S100A8/A9	S100 calcium-binding protein A8/A9
SHP-1/SHP-2	Src Homology 1/2
Siglec7	Sialic acid binding immunoglobulin-like lectin 7
Siglec9	Sialic acid binding immunoglobulin-like lectin 9;T regs, T regulatory cells
TACTILE	T cell activation, increased late expression
TGF-β	Transforming growth factor-β
TIGIT	T-cell immunoglobulin and ITIM domain
TIM3	T cell Immunoglobulin Domain and Mucin domain 3
TIMD4	T-cell immunoglobulin and mucin domain containing 4
TME	Tumor microenvironment
TNF-α	Tumor necrosis factor-α
TPD-L1	Tissue Programmed Death Ligand-1;TPS, Tumor proportional scores
ULBP-1/4	UL16 binding protein ¼.
