# Supplementary material for: Serum immune mediators as novel predictors of response to anti-PD-1/PD-L1 therapy in non-small cell lung cancer patients with high tissue-PD-L1 expression
Source: Front Immunol. 2023 May 15;14:1157100. doi: 10.3389/fimmu.2023.1157100 (PMC10225547; doi:10.3389/fimmu.2023.1157100)
Supplement: Supplementary file 1 [file Table_1.docx]

| **Soluble immune checkpoints/**  **circulating tumour antigens** | **Median (IQR)** |
| --- | --- |
|  |  |
| BTLA (pg/ml) | 451 ( 196-705) |
| CD137/4-1BB (pg/ml) | 61 ( 39-92) |
| CD152/CTLA4 (pg/ml) | 23 (4.0-38.2) |
| CD27 (pg/ml) | 1327 (556-1870) |
| CD28 (pg/ml) | 217 (55-334) |
| CD80 (pg/ml) | 97 (64-147) |
| GITR (pg/ml) | 14 (7-23) |
| IDO (pg/ml) | 36 (20-67) |
| LAG3 (pg/ml) | 108 (87-167) |
| PDL-1 (pg/ml) | 1.2 (0.14-3.43) |
| PDL-2 (pg/ml) | 1209 (650-2118) |
| PD-1 (pg/ml) | 39 (22-52) |
| TIM-3 (pg/ml) | 937 (566-1500) |
| Arginase (pg/ml) | 50 (31-57) |
| E-cadherin (pg/ml) | 1024 (58-6618) |
| MICA (pg/ml) | 93 (31-137) |
| MICB (pg/ml) | 38 (24-86) |
| NECTIN 2 (pg/ml) | 845 (602-1315) |
| NT5E (pg/ml) | 354 (179-604) |
| PVR (pg/ml) | 2555 (1779-3311) |
| SINGLEC 7 (pg/ml) | 318 (211-456) |
| SINGLEC 9 (pg/ml) | 64 (21-257) |
| ULBP 4 (pg/ml) | 282 (174-459) |
| OX40 (pg/ml) | 35 (28-55) |
| CD276 (pg/ml) | 11166 (1740-48061) |
| IAP (pg/ml) | 41 (28-63) |
| GALACTIN 9 (pg/ml) | 88 (54-124) |
| ICOS LIGAND (pg/ml) | 670 (257-1033) |
| S100 A8/A9 (pg/ml) | 2577 (1535-5553) |
| TIMD4 (pg/ml) | 681 (396-1911) |
| VISTA (pg/ml) | 57 (45-105) |
| CA-15-3 (U/ml) | 13 (7.0-34) |
| CA-19-9 (U/ml) | 85 (40-179) |
| CEA (pg/ml) | 1042 (730-11374) |
| CA125 (U/ml) | 17 (7.0-54) |
| CYFRA21-1 (pg/ml) | 980 (482-1982) |

**Supplementary Table 1 (S1): Median (IQR) of tested soluble biomarkers**
