# Supplementary material for: Serum immune mediators as novel predictors of response to anti-PD-1/PD-L1 therapy in non-small cell lung cancer patients with high tissue-PD-L1 expression
Source: Front Immunol. 2023 May 15;14:1157100. doi: 10.3389/fimmu.2023.1157100 (PMC10225547; doi:10.3389/fimmu.2023.1157100)
Supplement: Supplementary file 2 [file Table_2.docx]

**Supplementary Table 2 (S2) : Median (IQR) values of soluble biomarkers inTPD-L1 groups**

| Soluble biomarkers | TPD-L1 <50% | TPDL-1>50% | P value |
| --- | --- | --- | --- |
| SIGLEC-7 | 458 (360-594) | 252 (180-375) | 0.011 |
| SIGLEC-9 | 277 (143-288) | 30 (17-76) | 0.003 |
| ULBP-4 | 735 (338-982) | 194 (153-359) | 0.008 |
| PD-L2 | 772 (538-2393) | 1597 (909-2486) | 0.015 |

| TPD-L1 >50% | | |  |
| --- | --- | --- | --- |
|  | **Responders** | **Non-Responders** |  |
| PD-L2 | 1130 (771-1601) | 2486 (1524-2884) | 0.008 |
| TIMD4 | 349 (227-701) | 791 (663-1424) | 0.040 |
| NECTIN-2 | 742 (354-986) | 2327 (1013-4290) | 0.012 |
| CEA | 720 (224-1042) | 9516 (2043-13984) | 0.024 |
