# Supplementary material for: Serum immune mediators as novel predictors of response to anti-PD-1/PD-L1 therapy in non-small cell lung cancer patients with high tissue-PD-L1 expression
Source: Front Immunol. 2023 May 15;14:1157100. doi: 10.3389/fimmu.2023.1157100 (PMC10225547; doi:10.3389/fimmu.2023.1157100)
Supplement: Supplementary file 3 [file Table_3.docx]

| Expression in responding patients on different treatment types | | | |
| --- | --- | --- | --- |
|  | *Anti-PD-1/PD-L1 (n=9)*  *Group 1* | *Chemo-immunotherapy (n=6)*  *Group 2* |  |
| PD-1 | 20 (12-42) | 56 (42-339) | 0.012 |
| Expression in non-responding patients on different treatment types | | | |
|  | *Anti-PD-1/PD-L1 (n=9)*  *Group 1* | *Chemo-immunotherapy (n=7)*  *Group 2* |  |
| S100A8/A9 | 3707 (1720-11633) | 1550 (720-1962) | 0.0084 |

**Supplementary Table 3 (S3): Median (IQR) values of soluble biomarkers in responding and non- responding patients on different treatment types**
