# Supplementary material for: Serum immune mediators as novel predictors of response to anti-PD-1/PD-L1 therapy in non-small cell lung cancer patients with high tissue-PD-L1 expression
Source: Front Immunol. 2023 May 15;14:1157100. doi: 10.3389/fimmu.2023.1157100 (PMC10225547; doi:10.3389/fimmu.2023.1157100)
Supplement: Supplementary file 4 [file Table_4.docx]

**Supplementary Table 3 (S3): Median (IQR) values of soluble biomarkers between responders and non-responders irrespective of treatment types**

| Soluble biomarkers | Responders (n= 15) | Non-Responders (n= 16) | P value |
| --- | --- | --- | --- |
| CD80 | 77 (54-99) | 112 (79-254) | 0.023 |
| TIMD4 | 491 (283-1433) | 912 (575-2679) | 0.033 |
| CEA | 720 (275-1042) | 2013 (880-14028) | 0.008 |

*Concentration units: pg/ml*
